# Translation, cultural adaptation and linguistic validation of the postgraduate hospital educational environment measure into Arabic

**DOI:** 10.1186/s12909-024-05611-y

**Published:** 2024-06-05

**Authors:** Ghaith Alfakhry, Khattab Mustafa, Rawan Khwanda, Mervat Alhaffar, Khaled Alhomsi, Rama Kodmani

**Affiliations:** 1 Education Quality and Scientific Research Office, Al-Sham Private University, Damascus, Damascus Governorate, N/A, Baramekeh, Syria; 2 Program of Medical Education, Syrian Virtual University, Damascus Governorate, N/A, Damascus, Syria; 3 Department of Education, University of Oxford, 15 Norham Gardens, Oxford, OX2 6PY UK; 4 Department of Endodontics and Restorative Dentistry, Faculty of Dental Medicine, Damascus University, Damascus Governorate, N/A, Damascus, Syria; 5 Department of Pediatrics, Damascus Hospital, Damascus Governorate, N/A, Damascus, Syria; 6 Department of Global Health and Development, London School of Hygiene & Tropical Medicine Faculty of Public Health and Policy, London, UK; 7 Syria Research Group, London School of Hygiene & Tropical Medicine, NUS Saw Swee Hock School of Public Health, London, UK; 8 Department of Biomedical Sciences, Al-Sham Private University, Damascus Governorate, N/A, Damascus, Syria; 9 University Hospital of Dermatology and Venereology, Damascus University, Damascus Governorate, N/A, Damascus, Syria

**Keywords:** Translation, Validation, Postgraduate hospital educational environment measure, PHEEM, Arabic language, Syria

## Abstract

**Background:**

Assessment of the clinical learning environment (CLE) is an essential step that teaching hospitals routinely undertake to ensure the environment is conducive, learning-oriented and supportive of junior doctors’ education. The Postgraduate Hospital Educational Environment Measure (PHEEM) is an internationally recognized tool for assessing the CLE with evidence of high reliability and validity. Translation of PHEEM into other languages such as Spanish, Japanese and Persian enabled wider adoption of the instrument in the world. However, in Syria and other Arabic countries, a validated Arabic translation of PHEEM is still not available, making it difficult to adopt it and use it in Arabic contexts. This study aims to translate and culturally adapt the PHEEM from English into Arabic.

**Methods:**

This study followed the structured translation and validation process guideline proposed by Sousa & Rojjanasrirat 2010. First, the PHEEM went through forward translation by three translators, then reconciled with the aid of a fourth translator. Afterwards, two professional bicultural and bilingual translators conducted back translation into English and compared it with the original version. This formed the Pre-final Version (PFV) which was then pretested for clarity on a sample of medical residents in Damascus, Syria. Following appropriate modifications, the PFV was sent to a panel of experts for a comprehensive review of language clarity and to assess content validity.

**Results:**

A total of thirty-five medical residents were recruited. Ten items with language clarity issues were identified and modified according to the elicited suggestions. Thereafter, the modified PFV was presented to ten subject experts who identified three items in need of revision. The item-content Validity Index (CVI) was over 0.78 for all of the 40 items; the calculated scale-CVI was 0.945.

**Discussion:**

This study provided the first linguistically valid Arabic translation of the widely used PHEEM inventory. The next step is to conduct a full psychometric analysis of the Arabic PHEEM to provide further evidence of validity and reliability.

**Supplementary Information:**

The online version contains supplementary material available at 10.1186/s12909-024-05611-y.

## Introduction

Measuring the learning environment (LE) is an essential component of curriculum development and management, as it has well-recognized importance for students’ success, satisfaction and character development [1]. In medical education research, the conceptualization of the learning environment is difficult for it is multi-layered, encompassing various physical, emotional and intellectual aspects [2], and this sophistication and complexity is increased when the learning environment overlaps with the workplace environment such as in the postgraduate teaching hospital settings [3]. The clinical learning environment (CLE) has three sub-concepts: clinical work, learning and environment [3].

One of the most widely used instruments to measure the CLE is PHEEM (Postgraduate Hospital Educational Environment Measure), which has been specifically designed and validated for the purpose of assessing the postgraduate clinical teaching for hospital-based junior hospitals [4, 5]. In comparison to DREEM (Dundee Ready Educational Environment Measure), which is an instrument tool designed to assess undergraduate educational climates at medical schools, PHEEM is considered more content relevant to resident doctors as the items are focused on unique aspects of hospital-based learning such as learning autonomy and supervisor-doctor relationship which are pivotal in clinical education [4].

PHEEM is known for its established validity and reliability [6]. Its broad applicability across various specialties sets it apart from instruments designed for specific specialties [7] such as ATEEM (Anesthetic Theater Educational Environment Measure) [6] and STEEM (Surgical Theater Educational Environment Measure) [7].

The PHEEM inventory has been translated and validated into many languages including Spanish, Danish, Greek, Japanese and Persian [8–12]. Up to writing this, a validated Arabic translation of PHEEM is not available [13]. While PHEEM has proven effective in diverse contexts, understanding its impact in the unique setting of Syria is pivotal to underscore the importance of implementing the PHEEM in clinical education systems in conflict affected contexts. The absence of a validated Arabic translation limits its applicability, especially considering the significant contextual differences [16].

Grasping the specific context of Syria is crucial to underscore the unique importance of implementing the PHEEM in its clinical education system. During the past decade, the Syrian conflict has affected all aspects of life. The destruction of healthcare facilities, the financial constraints along with the huge immigration waves of healthcare professionals rendered the Syrian health system incapable of adequately meeting the healthcare demands of the Syrian population [14]. The deficits of the Syrian healthcare system have clearly manifested during the cholera outbreak, COVID-19 pandemic and most recently the devastating earthquake [15–17]. Resident doctors who make the frontline of healthcare workforce are working under extremely challenging conditions where medical resources are severely lacking [18]. It is only logical to assume that these substandard conditions have adverse effects on patient care, learning outcomes and residents’ physical and mental well-being [1, 18–20]. In such conditions, the learning environment is expected to deteriorate. A previous report measuring the learning environment at undergraduate medical schools in Syria has demonstrated students’ negative perception of the learning environment as measured by DREEM (Dundee Ready Educational Environment Measure) [21]. Undergraduate clinical students’ perceptions of the learning environment were particularly negative, scoring 95.6 out of 200 on the DREEM scale. This finding highlights potential deficit in the CLE not only in the undergraduate stage but also the postgraduate where the CLE is most prominent; nevertheless, an evaluation of medical residents’ perception of the CLE at teaching hospitals in Syria has yet to be conducted.

Before a wider application of the PHEEM is possible in Syria and other Arab World countries, the translation and linguistic validation of PHEEM is necessary to ensure its clarity, relevance and suitability to each country context. The translation and validation of PHEEMwould offer clinical educators in Syrian teaching hospitals a valuable tool for assessing key aspects of the clinical learning environment. By identifying and addressing critical factors that influence medical residents’ learning and working conditions, this tool could significantly enhance the overall educational experience. Ultimately, such improvements are likely to have a positive impact on healthcare delivery in these settings. Achieving successful validation in this special context has the potential to improve postgraduate learning environment evaluation and medical education, offering insightful information about healthcare and educational practices in the area. Therefore, the aim of this study is to translate and cross-culturally validate PHEEM from English into Arabic based on the Syrian context.

## Materials & methods

This is a cross-sectional study that followed a quantitative approach. The study was approved by the Ethics Committee of Al-Sham Private University (no. 68,127) and was conducted between February and April 2023.

### Study design

This study followed the translation and adaptation guidelines of Sousa and Rojjanasrirat [22], which is seven-step validation process for translation and cross-cultural adaptation of instruments in healthcare research. The steps of the guideline are: (1) two independent forward translations into the target translation are made; (2) reconciliation of the two translations; (3) blind back translation; (4) reconciliation of the two back translations; (5) pilot testing; (6) preliminary psychometric testing; (7) full psychometric testing.

“Using processes one and two [26], the PHEEM was translated from English into Arabic, and then back into English. Then, utilizing stages three and four, cross-cultural validation was carried out [26]. In the fifth step, the clarity of instructions, response format, and sentence structure of items were assessed, followed by content validity evaluation utilizing an expert committee technique. In order to allow for a more thorough investigation, the pilot and complete psychometric analysis (steps 6 and 7) were deferred to a future study project. The decision to leave the psychometric analysis was done in accordance to Sousa and Rojjanasrirat (2011), which suggests that in a large project involving the translation and validation of a questionnaire, multiple studies are necessary (Sousa & Rojjanasrirat, 2011). Consequently, we structured this study to cover validation steps 1 through 5. These steps encompass a substantial amount of detailed work that is crucial to the translation process. Given the complexity and significance of these steps, we believe that they merit reporting as an independent study. This approach ensures a thorough and focused analysis, allowing subsequent studies to build upon a solid foundation for further validation and psychometric assessment. The entire translation and validation processes were completed over a span of two months, from February 2023 to April 2023.”

### Settings and population

The postgraduate medical education in Syria is divided into two main residency programs. One falls under the Ministry of Higher Education [23], and the other is supervised by the Ministry of Health [24]; each has their own independent medical centers and hospitals. Physicians are admitted into either of them based on their specialty preference and graduation grade. Both systems have the same training duration, workload and accommodation conditions. The residency duration varies between specialties which is usually 5–7 years. In addition, both systems have some hierarchical structure, and provide specialist supervision, scientific lectures and educational events on regular basis, nonetheless. medical centers affiliated with the Ministry of Higher Education are more structured. The Syrian Board Specialization Certificate is granted after the completion of either residency programs [25]. Additionally, the Ministry of Higher Education grants a Master’s Degree certificate which allows residents to apply for a PhD programs unlike Ministry of Health residents. The curriculum is where the two systems diverge most. Residents in the Higher Education program are required to complete yearly clinical and theoretical tests, and at the conclusion of their residency, they are expected to turn in a research paper in the form of a master’s dissertation. However, the Ministry of Health only requires passing two exams: the final one at the conclusion of the first year.

### Description of postgraduate hospital educational environment measure (PHEEM)

PHEEM is an inventory designed to gauge the clinical learning environment for postgraduate medical residents. This instrument has been first introduced in 2005 by Roff et al. [4] who developed PHEEM using a combination of grounded theory and Delphi process. The final product was a 40-item inventory subdivided into three sub-scales: perceptions of role autonomy, perceptions of teaching, and perceptions of social support [4]. Each item is scored on a 5-point scale: 0 for Strongly Disagree, 1 for Disagree, 2 for Uncertain, 3 for Agree, 4 for Strongly Agree. Four items (no. 7, 8, 11 and 13) are negatively phrased and should be coded in reverse [4]. The maximum cumulative PHEEM score for the 40 items is 160 indicating the ideal learning environment as perceived by respondents. For interpreting the PHEEM score, the following guide was proposed [4]:

- 0–40  very poor.
- 41–80  plenty of problems.
- 81–120  more positive than negative.
- 121–160 excellent.

### Translation process

**Fig. 1:**
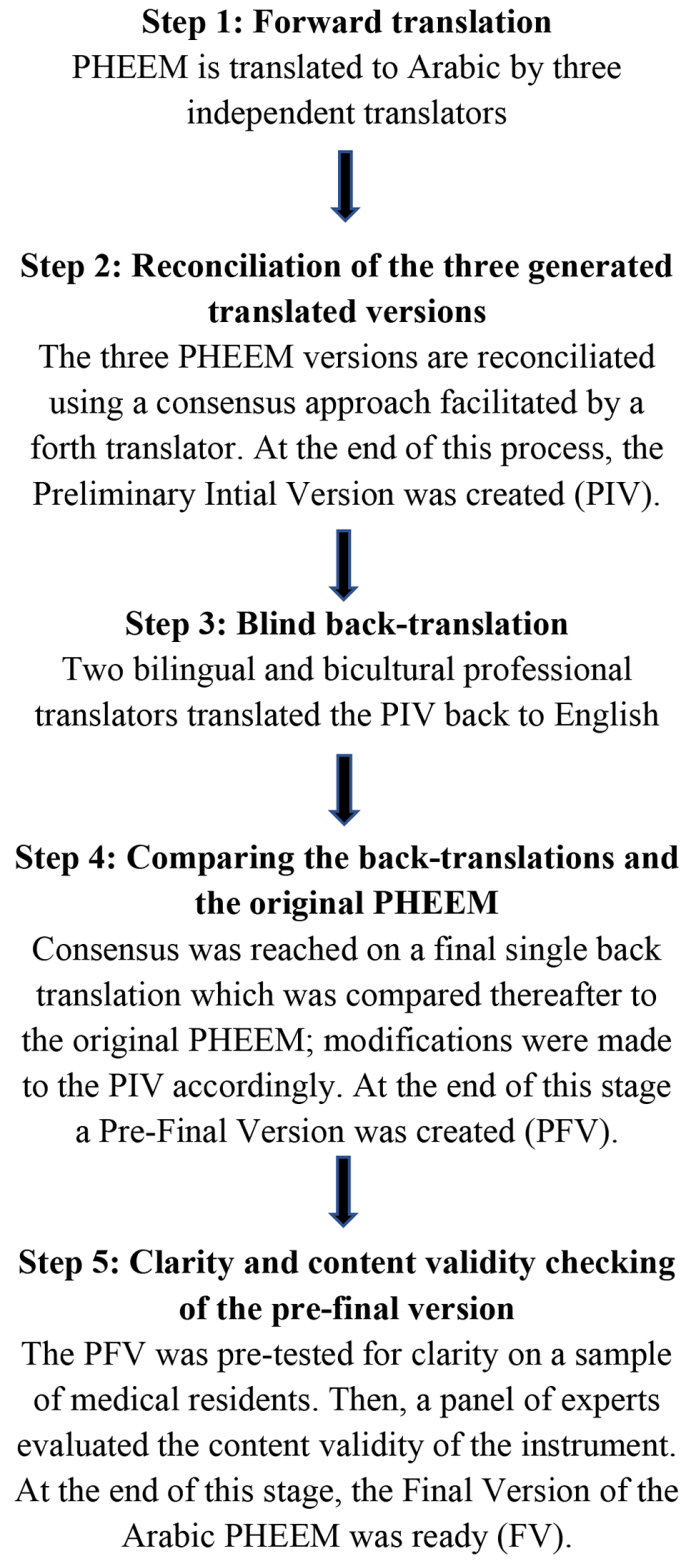
Summary of the process used in this study to translate and linguistically validate the PHEEM inventory into Arabic

This study followed a symmetrical translation approach in which equivalence is sought between the source language (SL; English) and target language (TL; Arabic). Symmetrical translation is not literal translation as it is faithful to meaning and colloquialism of both SL and TL [26]. A summary of the translation process steps is demonstrated in Fig. 1.

### Step 1: forward translation

Three independent experienced translators (KM, RK, RKh) were selected to conduct the forward translation. All translators’ mother’s tongue was Arabic (desired TL), and they were fluent in English. Distinct backgrounds were sought when selecting translators. The first translator, KM, was a dentist with qualification in medical education. The second and third translators were medical residents for the Ministry of Higher Education and Ministry of Health respectively. The second and third translators were familiar with the two main residency systems in Syria and had experience with the slang and jargon used within hospital settings. Selection of translators was done in a manner that makes the translation versions cover both the specialized terminology and colloquial language of hospital residents in Syria. Three Arabic PHEEMs were generated at the end of this stage.

### Step 2: reconciliation of the three generated translated versions

The three generated versions were compared by a fourth bilingual translator, GA, who identified ambiguities and discrepancies of semantics in each translation. A committee approach was used where all four translators discussed and resolved the identified discrepancies in the translations and consensus was reached in the translation. This generated the Preliminary Initial Version (PIV) of the Arabic PHEEM.

### Step 3: blind backwards translation

Two experienced bilingual and bicultural translators based in the UK were selected. Both translators were blind to the original instrument. The first translator, MA, demonstrated proficiency in healthcare terminology and the content area of the instrument, while the second translator, (DH), exhibited familiarity with the slang and colloquial language prevalent in the source language (SL).

### Step 4: comparison of the two back-translated versions

The two back-translated versions were compared to each other by a third translator (GA) and the two back translators, resulting in a consensus for the final back translation; thereafter, the final back translation was compared to the original PHEEM using a committee approach. The multidisciplinary committee included individuals from diverse backgrounds, such as healthcare, medical education, English literature, and learning sciences, bringing varied perspectives to the evaluation process. This diverse expertise ensured a comprehensive assessment of the back-translated version. The multidisciplinary committee included individuals from diverse backgrounds, such as healthcare, medical education, English literature, and learning sciences, bringing varied perspectives to the evaluation process. This diverse expertise ensured a comprehensive assessment of the back-translated version. For instance, healthcare professionals provided insights into the clinical relevance and applicability of the items, medical educators focused on the educational validity and pedagogical soundness, English literature experts ensured linguistic accuracy and cultural appropriateness, and learning scientists contributed an understanding of educational measurement and assessment principles. This variety of viewpoints was crucial for a thorough and accurate evaluation, as it allowed the committee to consider multiple facets of the translation, ensuring that the instrument maintained its integrity and relevance across different contexts.

Any discrepancies or ambiguities between the final back-translation and the original PHEEM were discussed among the committee members and resolved through consensus.

When discrepancies could not be resolved on any item or when items did not retain their original meaning, items were re-translated and back-translated (Step 1 through 4 were repeated on those items). The process was repeated until no discrepancies or ambiguities were detected when comparing the final back-translation with the original PHEEM. At the end of this step, the Pre-Final Version of the instrument in the TL (PFV) was ready.

### Step 5: clarity and content validity checking of the Arabic PHEEM

Participants from the target population (medical residents in Syria) were recruited using a paper-based and online survey developed using Google Forms. The recommended sample size was between 10 and 40 as per previous studies [27, 28]. Using convenience sampling, participants were recruited and were asked to rate the clarity of instructions, response format and items of the PFV using a dichotomous scale (clear, unclear). When selecting the “unclear”, participants were prompted to provide suggestions on how to rephrase and increase the clarity of the item. We established a minimum inter-rater agreement threshold of 80% for an item to be retained [29], i.e. 80% of participants rated the item as “clear”. Items failing to meet this 80% agreement criterion were subject to re-evaluation and the PFV was modified accordingly.

To further ensure the clarity and content validity of the PFV, an expert panel was used to calculate the Content Validity Index (CVI) in which ratings of relevance is provided, i.e. evaluation of relevance [30]. The panel also evaluated the clarity of the modified PFV [30]. Confirming these aspects is crucial as they ensure the instrument accurately measures the intended construct and that each item is clearly understood by respondents. This foundational step is vital for the reliability and validity of subsequent psychometric testing, as it underpins the overall quality and interpretability of the results. Without this initial verification, any further analysis could be based on an unstable foundation, potentially leading to misleading conclusions [22, 30].

The Content Validity Index (CVI) is a widely used statistical tool to quantify the validity of a test, survey, or questionnaire. It evaluates how well the items within the instrument represent the concept being measured. The process typically involves expert panel review of the items in the instrument using a rating scale for clarity and relevance; second, Item level CVI for each item is calculated by dividing the number of experts who rated the item as relevant by the total number of experts. For instance, if 8 out of 10 experts rate an item as relevant, the I-CVI for that item is 0.8. Thereafter, the scale level CVI is calculated by averaging the I-CVI for all items. Ten subject experts were chosen as per previous recommendations [22, 30]. The selection criteria of the panel of experts were: (1) being bicultural and bilingual, (2) clinical experience both in Syria and the UK, (3) experience and knowledge in medical education.

First, the expert panel evaluated the clarity of instructions and items of the modified PFV using a dichotomous scale (clear, unclear) and the minimum inter-rater agreement among the expert panel was set at 80% [29]. Thereafter, the expert panel evaluated the modified PFV for content-related validity on a 4-point scale (1 = not relevant, 2 = unable to assess relevance, 3 = relevant but needs minor alteration, 4 = very relevant). Then, the CVI at the item level (I-CVI) and at the scale level (S-CVI) were calculated. The S-CVI was measured by averaging calculations as it is preferred [31]. With a panel of ten experts, the acceptable values for I-CVI and S-CVI were set at a minimum of 0.78 [34] and 0.90 [36], respectively. Expert evaluations were collected using either face-to-face approach or electronically using an online questionnaire survey developed via Google Forms. This step was necessary to confirm and improve the clarity of modified PFV as well as to ensure the conceptual, semantic and content equivalency of the modified PFV prior to full psychometric testing.

Data were processed and analyzed using Microsoft Excel (2019) and IBM SPSS Statistics for Windows, version 26 (IBM Corp., Armonk, N.Y., USA). The electronic platform used to conduct online surveys was Google Forms.

## Results

A sample of 35 medical residents were recruited to evaluate the clarity of the PFV. Characteristics of respondents are shown in Table 1.

**Table 1:** Demographic data of the sample of residents used to evaluate the clarity of pre-final version of Arabic PHEEM

**Sex**	**Male**		**Female**			
	18		17			
University	Damascus University		Aleppo University		Tishreen University	
	26		3		2	
Specialization affiliation	Ministry of Higher Education		Ministry of Health		Other*	
	17		15		2	
Residency year	1st	2nd	3rd	4th	5th	Specialists
	3	9	6	5	6	4
Age	Mean ± SD					
	27.7 ± 2.1					

*Ministry of Defense or Police Department residents

The instructions and response format were rated as clear by all participants (*n* = 35). As for the items, thirty-five items (87.5%) were rated as clear by at least 80% of the sample. Only five items were scored below this cut-off point and therefore were re-evaluated and modified according to the suggestions provided by participants. Further, items that were scored as clear by 80–85% (Table 2) of the sample were also re-evaluated and modified to increase clarity as much as possible.

**Table 2:** Clarity evaluation of the pre-final version of PHEEM inventory on a sample from the target population (*n* = 35)

Rated as clear by	Number of items (%)
95–100% of the sample	8 (20%)
90–95% of the sample	18 (45%)
85–90% of the sample	4 (10%)
80–85% of the sample	5 (12.5%)-rephrased
< 80% of the sample	5 (12.5%)-rephrased

After re-evaluation, a modified PFV (Table 5s, Supplementary material I) was generated and then pretested for clarity and relevance by a panel of ten experts. Characteristics of the panel of experts can be found in Table 3. A summary of experts’ evaluations of the clarity of items is presented in Table 4. Only three items were evaluated as “unclear” by less than eight experts. Four items were evaluated as “clear” by eight experts and the rest of the items (33) were evaluated as clear by nine or all ten experts.

**Table 3:** Characteristics of the panel of experts selected for pilot pretesting and content validity checking

Sex	Age	Specialty	hospital/university	Experience in medical education
Male	28	Internal Medicine	University Hospital Geelong, Australia	Yes
Male	36	Dermatology	Damascus University, Syria	Yes
Male	44	GIM - Cardiology	King’s College Hospital, UK	Yes
Male	53	Fixed Prosthodontics	Newcastle University, UK	Yes
Female	51	Preventive medicine	University of Glasgow, UK	Yes
Female	62	Dermatology	King’s College London, UK	Yes
Female	29	Neurology	University College London, UK	Yes
Male	32	Trauma and Orthopedics, Medical Education	Imperial College London, UK Dundee University, UK	Yes
Male	26	Public Health	University of Glasgow, UK	Yes
Male	32	Cardiology	Tishreen University, Syria	Yes

*Nationality of all experts is Syrian*

**Table 4:** Clarity evaluation of the *modified* Pre-Final Version (PFV) of the Arabic PHEEM inventory (40 items) on a panel of experts (*n* = 10)

Rated as clear by	Number of items (%)
All ten experts	26 (65%)
Nine experts	7 (17.5%)
Eight experts	4 (10%)
< Eight experts	3 (7.5%)

In Table 5s, Supplementary material I, the content validity index of the modified PFV at the item level (I-CVI) as rated by experts is presented, and evaluation of the clarity of items is also illustrated. The calculated scale-CVI for the 40 items was 0.945.

Seven items had been marked as unclear by 2 or more experts and these were: item no. 2, 3, 5, 18, 24, 25, 33). These items (except for 25) went through minor revision according to the expert feedback. Eight experts marked the item no 25 clear, and consensus on not changing the translation was reached by the research team and translators.

All corrections made to the modified PFV are illustrated in Table 6s, supplementary material I. Moreover, in item no. 38 the Arabic translation for (junior doctors who fail to complete their training satisfactorily) was substituted with a different Arabic term that is more polite and acceptable. The final version of the translated PHEEM is available in supplementary material II.

## Discussion

This study was set out to translate and culturally adapt the Postgraduate Hospital Educational Environment Measure (PHEEM) from English into Arabic. To this end, a rigorous translation process was conducted. This multi-step sophisticated translation process produced the Pre-Final Version (PFV) of the questionnaire in the target language (Arabic). Subsequently, the PFV was pretested for clarity on a sample from the target population (medical residents in Syria) and was then modified according to the suggestions provided by the respondents. The modified PFV was presented to a panel of deliberately selected subject experts who evaluated the clarity and content validity of the instructions and items. All items achieved an acceptable I-CVI and the total scale achieved an acceptable S-CVI. As for clarity evaluation, experts identified seven items lacking in clarity which were thereafter, modified as per the expert panel’s recommendations.

Cultural adaptation in terms of considering cultural nuances and contextual factors were considered during the translation process. A case in point is the translation of item number 17, “My hours conform to the New Deal;” the New Deal is specifically referenced in the context of the United Kingdom and refers to the set of reforms and policies introduced to improve the work conditions; one central component of the New Deal is the reduction of working hours for junior doctors. The literal translation of the New Deal would make no sense in our context, therefore the translation was changed to convey the idea that working hours at the hospital are appropriate. Other instances where cultural differences appeared were in items related to the issue of racism, induction program, and paging doctors (items 4, 7, 11). In Syria, international students are very scarce in comparison to the UK, so issues of racism are not applicable to the Syrian context. Secondly, induction programs are not usually held officially as part of the medical residency program. Lastly, in item number 11 “I am bleeped inappropriately” does not apply to the Syrian context, as hospitals do not use bleeps (or pagers) to alert the clinical staff. Instead, mobile phones are used. Overall, the translation thoughtfully considered the cultural nuances in the translation; relevance and clarity evaluations in our study are evidence for the cultural relevance of Arabic PHEEM.

The PHEEM inventory has been proved to be a valid and reliable instrument in various countries and languages [8–12]. In the Arab World, PHEEM was applied mostly in its original English version [13] which has been shown to be reliable and valid in the Arabic context according to one study [32]. Another study in Saudi Arabia reported the use of both the English version and Arabic translation of the PHEEM [33]; however, the Arabic translation was not made publicly available and more interestingly, almost all participants (97%) preferred the use of Arabic version over the English version [33]. This demonstrates the importance of translating and cross-culturally adapting PHEEM when applying it in an Arabic context. Similarly, one of the constraints of the PHEEM study conducted in Saudi Arabia was that there was no official Arabic translation of the PHEEM [13]. This highlights the necessity of translating and adapting the PHEEM into the Arabic language.

The Syrian context is different from other Arab countries. Syria has been going through civil-war for over 12 years now. The civil war has led to severe resource shortages, including essential medical supplies and equipment [34, 35]. For example, residents often lack access to basic items like gloves and surgical masks, compromising infection control standards. Additionally, major shortage in teaching staff has also affected residents negatively [34, 35]. The economic situation has further exacerbated these challenges, resulting in poor living conditions for residents. Many dormitories suffer from low hygiene standards, which negatively impact the residents’ physical and mental well-being, thereby affecting their learning and performance [34]. Moreover, these conditions contribute to a high dropout rate among medical residents [34–36]. These differences mandate the addition of evaluation of available resources to the PHEEM questionnaire in order to correctly assess the learning environment in under-resourced settings. Moreover, the psychological health of Syrian residents must be taken into consideration especially in war-torn contexts like Syria **where residents may experience trauma, chronic stress, and anxiety due to the ongoing conflict and its aftermath. The continuous exposure to high work pressure, increasing violence, resource scarcity, and inadequate living conditions significantly impacts their mental well-being, leading to increased rates of burnout, depression, and other psychological issues.** [20, 34]. Therefore, the authors recommend the addition of two domains: the *perception of available resources* which should be designed to measure the available hospital resources and investigate how it affects working and learning; secondly, the *perception of psychological support* which should be designed to evaluate the psychological burden of working in under-resourced and chaotic hospital setting in a war-ravaged country like Syria. Availability of resources affects residents ability to practice medicine effectively and henceforth learning on the job especially in an age where medical technology and equipment are fundamental for everyday practice; although literature of the effect of lack of resources on teaching hospitals in Syria are scarce, recent papers has highlighted the effect of war and resource availability on medical students’ performance [37] and their perception of the learning environment [21, 38, 39]; some residents can miss out the chance to practice essential procedural skills for this reason [34]. As for the psychological health domain, studies have numerously showed the negative effect of war on psychological health of residents and having items to address, assess and bring forward this important area in our vulnerable context is very important from an educational perspective as well as wellbeing perspective [20, 40, 41]. A qualitative approach may also be useful in exploring the depths and specific nuances of each hospital setting.

A study in 2022 used the DREEM inventory to evaluate the learning environment at major medical schools in Syria [21]; students in the clinical stage (*n* = 1008) reported a more negative perception of the educational environment (DREEM = 95.6/200) than their pre-clinical counterparts. Perception of learning and social support were especially negative, scoring 41% and 42% respectively [21]. These two domains in the DREEM questionnaire closely relate to the perception of teaching and perceptions of social support in the PHEEM inventory. The authors predict that the postgraduate learning environment at teaching hospitals in Syria will show more significant shortcomings compared to the learning environment in the undergraduate stage. This prediction is based on the findings of a previous study that investigated the clinical learning environment for postgraduate dental students at Damascus University [35]. In the previous report, faculty inaccessibility and negligence of their teaching duties emerged as major themes [35]. Further, lack of peer support, under-resourced facilities and excessive workload were all aspects that characterized the clinical learning environment for postgraduate dental students [35]. To conclude, medical residents in Syria are facing significant challenges due to certain dimensions of the clinical learning environment; accurate assessment of thereof is necessary to raise awareness to these problems and design appropriate interventions. It could also be possible that the application of PHEEM in the Syrian context where less favorable working/learning conditions are present may show a *floor effect* (high percentage of participants scoring the minimum score) and thus decrease the discriminatory value of the tool in this specific context. Further studies are necessary to check this hypothesis.

## Conclusion

This study provided the first linguistically validated Arabic translation of the widely used PHEEM inventory. Evidence of language clarity and content relevance of the Arabic PHEEM to the hospital settings in Syria were established. However, this study did not provide evidence of reliability or construct validity of the Arabic PHEEM. A full psychometric testing of the Arabic PHEEM is still necessary to provide further evidence of validity and reliability; hence, enabling wider adoption within the Arab World.

This study makes a major contribution to the medical education literature being the first study to provide a validated Arabic translation of the PHEEM inventory; this could encourage a larger adoption of the instrument in the twenty-two Arabic speaking countries. Researchers and curriculum developers will be better equipped to identify areas of improvement in hospital learning environment and therefore, be able to recommend appropriate interventions which could optimize the experience and performance of medical professionals and ultimately improve the provision of healthcare for patients.

This study has several strengths such as the use of a rigorous guideline for translation and validation [22]. Further, this study has attempted to pilot pretest the translated version on a sample from the target population and improved clarity accordingly. Additionally, the content validity of the questionnaire was also checked and confirmed by a panel of experts.

When applying the Arabic PHEEM in contexts that are significantly affected by war like Syria, it might be possible to have a floor effect (i.e. significant portion of participants scoring on the lower end of the scale), therefore undermining its discriminant validity and sensitivity to detect finer variations, potentially masking areas that require more attention and hindering targeted educational interventions. Exploration of this point is important for future studies using PHEEM in war-ravaged contexts like Syria.

A limitation of this study is that it cannot fully ensure the clarity of the language of the produced Arabic PHEEM in other Arabic countries which might have different health systems and colloquial terminology. Cross-cultural adaptation of instruments like the PHEEM in Arabic-speaking countries involves several challenges. Firstly, linguistic diversity and regional dialects can lead to variations in the interpretation of questions and responses, affecting the tool’s uniformity and reliability. Secondly, cultural differences in medical education and practice between countries can influence how certain concepts are understood and valued. For studies aiming to cross-culturally validate the Arabic PHEEM, it is recommended to engage a diverse panel of experts from various Arabic-speaking regions to ensure the tool’s relevance and sensitivity to regional nuances. Incorporating a pilot study phase across different countries can help identify and address specific cultural and linguistic discrepancies. Additionally, employing qualitative methods like focus groups and interviews can provide deeper insights into the cultural contexts and potential modifications needed for effective cross-cultural validation.

Another limitation was that the final version of the Arabic PHEEM was not sent back to experts to confirm and accept the modifications. The decision to not resend the final version of the Arabic PHEEM to the experts was partly due to the challenges we encountered in re-establishing contact with all the experts within our project timeline. Additionally, the modifications made after eliciting expert feedback were minimal and only entailed minor linguistic revision for seven items, not altering the substantive content. Therefore, a comprehensive internal consensus process, involving experienced bilingual medical education experts was relied upon to ensure the validity and integrity of the final version.

### Electronic supplementary material

Below is the link to the electronic supplementary material.

Supplementary Material 1

Supplementary Material 2

## Data Availability

The datasets used and/or analysed during the current study are available from the corresponding author upon reasonable request.
