# Supplementary material for: Translation, cultural adaptation and linguistic validation of the postgraduate hospital educational environment measure into Arabic
Source: BMC Med Educ. 2024 Jun 5;24:625. doi: 10.1186/s12909-024-05611-y (PMC11154972; doi:10.1186/s12909-024-05611-y)
Supplement: Supplementary file 1 — Supplementary Material 1 [file 12909_2024_5611_MOESM1_ESM.docx]

**Table 5s**. Clarity evaluation and item-Content Validity Index (I-CVI) of the modified Pre-Final Version of the Arabic PHEEM using a panel of experts (n=10).

| Item | Clear | I-CVI |
| --- | --- | --- |
| 1.I have a contract of employment that provides information about hours of work  (لدي عقد عمل يبين عدد ساعات الدوام) | 10 | 1 |
| 2.My clinical teachers set clear expectations  (يحدد الاختصاصيون المتطلبات المرجوة مني) | 8 | 0.9 |
| 3.I have protected educational time in this post  (الوقت المخصص للتعلم ذو أهمية بالغة في المشفى) | 8 | 0.9 |
| 4.I had an informative induction programme  (تلقيت جلسة تعريفيّة بمهامي و ببروتوكولات العمل) | 10 | 0.9 |
| 5.I have the appropriate level of responsibility in this post  (يتناسب مستوى الواجبات المطلوبة مني مع دوري كطبيب مقيم) | 7 | 0.9 |
| 6.I have good clinical supervision at all times  (أخضع لإشراف سريري جيد طول الوقت) | 10 | 1 |
| 7.There is racism in this post  (يوجد تمييز على أساس -المنشأ, الدين, الحالة الاجتماعية, العمر- في عملي) | 9 | 0.8 |
| 8.I have to perform inappropriate tasks  (علي القيام بمهام لا تليق بدوري كطبيب مقيم) | 9 | 0.8 |
| 9.There is an informative Junior Doctors handbook  (تم تقديم كتيب إرشادات شامل للمعلومات الضرورية للأطباء المستجدين) | 10 | 1 |
| 10.My clinical teachers have good communication skills  (أرى أن الاختصاصيين يتمتعون بمهارات تواصل جيدة) | 10 | 1 |
| 11.I am bleeped inappropriately  (يتم استدعائي لأمور غير ضرورية أثناء المناوبة) | 9 | 0.8 |
| 12.I am able to participate actively in educational events  (أتمكن من المشاركة بشكل تفاعلي في الفعاليات التعليمية) | 10 | 1 |
| 13.There is sex discrimination in this post  (يوجد تمييز على أساس الجنس في عملي) | 10 | 1 |
| 14.There are clear clinical protocols in this post  (يوجد بروتوكولات سريرية محددة في هذا المشفى) | 10 | 0.9 |
| 15.My clinical teachers are enthusiastic  (أرى أن الاختصاصيين يتمتعون بشغف للتعليم) | 10 | 1 |
| 16.I have good collaboration with other doctors in my grade  (أتعاون جيدا مع زملائي) | 10 | 0.9 |
| 17.My hours conform to the New Deal  (عدد ساعات عملي في المشفى مقبول) | 10 | 0.9 |
| 18.I have the opportunity to provide continuity of care  (تتاح لي فرصة متابعة الحالة الصحية لمرضاي) | 8 | 0.9 |
| 19.I have suitability access to career advice  (يقدم الأخصائيون المشورة بخصوص مستقبلي المهني) | 10 | 1 |
| 20.This hospital has good quality accommodation for junior doctors, especially when on call  (تؤمن المشفى إقامة جيدة للأطباء المستجدين ولاسيما أثناء المناوبة) | 10 | 1 |
| 21.There is access to an educational programme relevant to my needs  (المنهاج متوافق مع احتياجاتي التعلمية) | 10 | 0.9 |
| 22.I get regular feedback from seniors  (أتلقى ملاحظات تعليمية بشكل دوري من الأطباء في السنوات الأعلى) | 10 | 0.9 |
| 23.My clinical teachers are well organized  (يؤدي الأخصائيون مهامهم بشكل منظم) | 10 | 1 |
| 24.I feel physically safe within the hospital environment  (لا أشعر بالأمان الفيزيائي في بيئة المشفى) | 6 | 1 |
| 25.There is a no-blame culture in this post  (لا توجد ثقافة "إلقاء اللوم" عند حدوث خطأ ما) | 8 | 0.8 |
| 26.There are adequate catering facilities when I am on call  (تتوفر مرافق إطعام جيدة أثناء المناوبة) | 10 | 1 |
| 27.I have enough clinical learning opportunities for my needs  (يوجد فرص كافية من الممارسة السريرية لتلبية احتياجاتي التعلمية) | 10 | 1 |
| 28.My clinical teachers have good teaching skills  (يتمتع الأخصائيون بمهارات تدريسية جيدة) | 10 | 1 |
| 29.I feel part of a team working here  (أشعر أنني جزء من فريق عمل هنا) | 10 | 1 |
| 30.I have opportunities to acquire the appropriate practical procedures for my grade  (لدي فرص لتعلم الإجراءات العملية الملائمة لسنتي الدراسية) | 10 | 1 |
| 31.My clinical teachers are accessible  (التواصل مع الاختصاصيين يسير) | 9 | 1 |
| 32.My workload in this job is fine  (عبء العمل مقبول) | 9 | 0.9 |
| 33.Senior staff utilize learning opportunities effectively  (ينتهز الأطباء الأعلى مرتبةً فرص التعلم لإفادتي) | 5 | 1 |
| 34.The training in this post makes me feel ready to be a SpR/Consultant  (أشعرأن تدريبي السريري يعدني للممارسة كطبيب اختصاصي) | 9 | 1 |
| 35.My clinical teachers have good mentoring skills  (يتمتع الاختصاصيون بقدرة جيدة على الإرشاد) | 10 | 1 |
| 36.I get a lot of enjoyment out of my present job  (أستمتع للغاية بعملي الحالي) | 10 | 0.9 |
| 37.My clinical teachers encourage me to be an independent learner  (يشجعني الاختصاصيون على التعلم المعتمد على الذات) | 9 | 1 |
| 38.There are good counselling opportunities for junior doctors who fail to complete their training satisfactorily. (يوفر المشفى دورات خاصة لدعم الأطباء الراسبين) | 10 | 0.8 |
| 39.The clinical teachers provide me with good feedback on my strengths and weaknesses. (يقدم الاختصاصيون ملاحظات قيمة حول نقاط القوة والضعف لدي) | 10 | 1 |
| 40.My clinical teachers promote an atmosphere of mutual respect  (يخلق الاختصاصيون جوّاً من الاحترام المتبادل) | 10 | 1 |

**Table 6s.** Editing made to the modified PFV after expert panel feedback

| **Final translation** | **Pre-final version** | **Item no.** |
| --- | --- | --- |
| يحدد الاختصاصيون مسؤوليات العمل المطلوبة مني/  Specialists set the job responsibilities required of me | يحدد الاختصاصيون المتطلبات المرجوة مني  My clinical teachers set clear expectations | 2 |
| الوقت المخصص للعملية التعليمية ذو أهمية بالغة ضمن المشفى/Time dedicated for the educational process is of utmost importance in the hospital | الوقت المخصص للتعلم ذو أهمية بالغة في المشفى/Time dedicated for learning is of utmost importance in the hospital | 3 |
| يتناسب مستوى المهام المطلوبة مني مع سنتي الدراسية  Level of responsibilities is appropriate to my year of study | يتناسب مستوى الواجبات المطلوبة مني مع دوري كطبيب مقيم/  I have the appropriate level of responsibility in this post | 5 |
| تتاح لي فرصة متابعة الرعاية لمرضاي بعد تقديم الخدمة الطبية  I have the opportunity to provide continuous healthcare after providing the health service | تتاح لي فرصة متابعة الحالة الصحية لمرضاي  I have the chance to follow up the health condition of my patients | 18 |
| أشعر بالامان في بيئة المشفى  I feel safe in the hospital environment | لا أشعر بالأمان الفيزيائي في بيئة المشفى  I do not feel physically safe in the hospital environment | 24 |
| يغتنم الأطباء الأعلى مرتبة الفرص لتعليمي  Senior doctors make use of opportunities to teach me | ينتهز الأطباء الأعلى مرتبةً فرص التعلم لإفادتي  Senior doctors exploit learning opportunities to benefit me | 33 |
| يوفر المشفى دورات خاصة لدعم الأطباء المتعثرين دراسيا  The hospital provides special courses to support doctors who are falling behind academically | يوفر المشفى دورات خاصة لدعم الأطباء الراسبين  The hospital provides special courses to support doctors who failed their year | 38 |
