# Supplementary material for: Translation, cultural adaptation and linguistic validation of the postgraduate hospital educational environment measure into Arabic
Source: BMC Med Educ. 2024 Jun 5;24:625. doi: 10.1186/s12909-024-05611-y (PMC11154972; doi:10.1186/s12909-024-05611-y)
Supplement: Supplementary file 2 — Supplementary Material 2 [file 12909_2024_5611_MOESM2_ESM.pdf]

مقياس البيئة التعليمية في المشافي للأطباء المقيمين
Postgraduate Hospital Educational Environment Measure

الجنس/Sex : ☐ ذكر /male ☐ أنثى / female

العمر/age: ....

الاختصاص/specialty:..

المفاضلة المقبول فيها للاختصاص/accepted for specialty at :

- ◻ تعليم عالي/Ministry of Higher Education
- ◻ وزارة الصحة/Ministry of Health
- ◻ وزارة الدفاع/Ministry of Defense
- ◻ أخرى/ Other

سنة الدراسة الحالية/Current Year of study:

☐ 1 ☐ 2 ☐ 3 ☐ 4 ☐ 5 ☐ 6 ☐ 7 ☐ امتياز

الجامعة المتخرج منها/University you studied at:...

المعدل الدراسي الحالي لك كطبيب مقيم (علامة فحص البورد لطلاب الصحة)/ current overall score or board exam score for Ministry of Health specialists: ....%

المشفى المقيم فيه حاليا/Hospital you working at currently:..

المشافي التي اقمت فيها سابقا/ Hospitals you worked at before :...................................

تتعلق البنود التالية جميعها بتجربتك الحالية خلال تخصصك. الرجاء قراءة كل عبارة وتقييمها بما يتناسب مع شعورك اتجاه دورك الحالي كمقيم في المشفى/المشافي التي تداوم فيها. الأمر يتعلق بمنظورك و تجربتك

Supplementary material II: The final version of the translated PHEEM

The following items all relate to your current /الشخصية في اطار دورك الحالي في المشفى experience in your specialty. Please read each statement and rate it as it applies to your own feelings about your present position in this hospital. It is about your personal perceptions of the current post.

لا أوافق بشدة<br>Strongly disagree	لا أوافق<br>Disagree	غير متأكد<br>Unsure	أوافق<br>Agree	أوافق بشدة<br>Strongly agree	ضع إشارة في المربع المناسب	
					لديّ عقد عمل يبين عدد ساعات الدوام<br>I have a contract of employment that provides information about hours of work	1
					يحدد الاختصاصيون مسؤوليات العمل المطلوبة مني<br>Specialists set the job responsibilities required of me	2
					الوقت المخصص للعملية التعليمية ذو أهمية بالغة ضمن المشفى<br>Time dedicated for the educational process is of utmost importance in the hospital	3
					تلقيت جلسة تعريفيّة بمهامي و بروتوكولات العمل<br>I had an informative induction programme	4
					يتناسب مستوى المهام المطلوبة مني مع سنتي الدراسية<br>Level of responsibilities is appropriate to my year of study	5
					أخضع لإشراف سريريّ جيد طول الوقت<br>I have good clinical supervision at all times	6
					يوجد تمييز على أساس (المنشأ، الدين، الحالة الاجتماعية، العمر) في عملي<br>There is discrimination based on (Ethnicity, religion, socioeconomic status, age) in this post	7
					عليّ القيام بمهام لا تليق بدوري كطبيب مقيم<br>I have to perform inappropriate tasks	8

Supplementary material II: The final version of the translated PHEEM

لا أوافق بشدة<br>Strongly disagree	لا أوافق<br>Disagree	غير متأكد<br>Unsure	أوافق<br>Agree	أوافق بشدة<br>Strongly agree		
					تم تقديم كتيّب إرشادات شامل للمعلومات الضرورية للأطباء المستجدين<br>There is an informative Junior Doctors handbook	9
					أرى أن الاختصاصيين يتمتعون بمهارات تواصل جيدة<br>My clinical teachers have good communication skills	10
					يتم استدعائي لأمور غير ضرورية أثناء المناوبة<br>I am being summoned for unnecessary matters when I am on call	11
					أتمكن من المشاركة بشكل تفاعلي في الفعاليات التعليمية<br>I am able to participate actively in educational events	12
					يوجد تمييز على أساس الجنس في عملي<br>There is sex discrimination in this post	13
					يوجد بروتوكولات سريرية محددة في هذا المشفى<br>There are clear clinical protocols in this post	14
					أرى أن الاختصاصيين يتمتعون بشغف للتعليم<br>Specialists have passion for education	15
					أتعاون جيداً مع زملائي<br>I have good collaboration with other doctors	16
					عدد ساعات عملي في المشفى مقبول<br>Number of working hours at the hospital is acceptable	17
					تتاح لي فرصة متابعة الرعاية لمرضاي بعد تقديم الخدمة الطبية<br>I have the opportunity to provide continuous healthcare after providing the health service	18
					يقدم الاختصاصيون المشورة بخصوص مستقبلي المهني<br>Specialists provide advice regarding my professional career	19
					تؤمن المشفى إقامة جيدة للأطباء المستجدين ولاسيما أثناء المناوبة<br>This hospital has good quality accommodation for junior doctors, especially when on call	20

Supplementary material II: The final version of the translated PHEEM

لا أوافق بشدة<br>**Strongly disagree**	لا أوافق<br>**Disagree**	غير متأكد<br>**Unsure**	أوافق<br>**Agree**	أوافق بشدة<br>**Strongly agree**		
					المنهاج متوافق مع احتياجاتي التعلمية<br>The curriculum is appropriate to meet my educational needs	21
					أتلقى ملاحظات تعليمية بشكل دوري من الأطباء المتقدمين عليّ بسنوات<br>. I get regular feedback from senior doctors	22
					يؤدي الاختصاصيون مهامهم بشكل منظم<br>Specialists perform their tasks in an organized manner	23
					أشعر بالأمان في بيئة المشفى<br>I feel safe in the hospital environment	24
					لا توجد ثقافة "إلقاء اللوم" عند حدوث خطأ ما<br>There is a no-blame culture in this post	25
					تتوفر مرافق إطعام جيدة أثناء المناوبة<br>There are adequate catering facilities when I am on call	26
					يوجد فرص كافية من الممارسة السريرية لتلبية احتياجاتي التعلمية<br>I have enough clinical learning opportunities for my needs	27
					يتمتع الاختصاصيون بمهارات تدريسية جيدة<br>My clinical teachers have good teaching skills	28
					أشعر أنني جزء من فريق عمل في هذا المشفى<br>I feel part of a team working here	29
					لدي فرص لتعلم الإجراءات العملية الملائمة لسنتي الدراسية<br>I have opportunities to acquire the appropriate practical procedures for my grade	30
					التواصل مع الاختصاصيين يسير<br>Specialists are accessible	31
					عبء العمل مقبول<br>My workload in this job is fine	32
					يغتنم الأطباء الأعلى مرتبة الفرص لتعليمي<br>Senior doctors make use of opportunities to teach me	33

Supplementary material II: The final version of the translated PHEEM

لا أوافق بشدة Strongly disagree	لا أوافق Disagree	غير متأكد Unsure	أوافق Agree	أوافق بشدة Strongly agree		
					أشعرأن تدريبي السريري يُعدُّني للممارسة كطبيب اختصاصي<br>The training in this post makes me feel ready to be a SpR/Consultant	34
					يتمتع الاختصاصيون بقدرة جيدة على الإرشاد<br>Specialists have good mentoring skills	35
					أستمتع للغاية بعملي الحالي<br>I get a lot of enjoyment out of my present job	36
					يشجعني الاختصاصيون على التعلم المعتمد على الذات<br>Specialists encourage me to be an independent learner	37
					يوفر المشفى دورات خاصة لدعم الأطباء المتعثرين دراسياً<br>The hospital provides special courses to support doctors who are falling behind academically	38
					يقدم الاختصاصيون ملاحظات قيمة حول نقاط القوة والضعف لديّ<br>Specialists provide me with good feedback on my strengths and weaknesses.	39
					يخلق الاختصاصيون جوّاً من الاحترام المتبادل<br>Specialists promote an atmosphere of mutual respect	40

هل لديك ما تود إضافته بخصوص بيئة العمل السريرية في المشفى الذي تعمل فيه ؟

Do you have anything you would like to add regarding the clinical working environment at the hospital you work in?

....

....

....
